# Supplementary material for: First Indications of Omsk Haemorrhagic Fever Virus beyond Russia
Source: Viruses. 2022 Apr 4;14(4):754. doi: 10.3390/v14040754 (PMC9030969; doi:10.3390/v14040754)
Supplement: Supplementary file 1 [file viruses-14-00754-s001.zip › viruses-1654709-supplementary.pdf]

Supplement Table S1.: Overview of tick collection, sampling sites, captured numbers and numbers of positive specimen.

Region	Year	sampling region	Sampling site	Tickpool Total	*D. marginatus* (tickpool)	*D. reticulatus* (tickpool)	*D. niveus* (tickpool)	*I. persulcatus* (tickpool)	OHFV positive of sampling site n (%)	OHFV in species	n OHFV positive	(%)	MIR
Akmola	2016	Sandyktau region	village Sandyktau	90	81	2	5	2	1(1.1)	*D. marginatus*	**1**	**1,1**	0,01
	2018	Sandyktau region	village Novonikolskoye	23	0	22	0	1	0		0	0,0	
			village Sandyktau	39	9	29	0	1	5(13.2)	*D. reticulatus*	**5**	**12,8**	0,17
		Zerendy region	village Sadovoye	66	0	64	0	2	2(3.0)	*D. reticulatus, I. persulcatus*	**2 (1 +1)**	**3,0 (1.5 + 1.5)**	0.02 0.50
	2019	Ayrtau region	village Imantau	60	24	35	0	1	0		0	0,0	
		Burabay region	village Katarkol	46	36	9	0	1	0		0	0,0	
		Sandyktau region	village Novonikolskoye	106	89	15	0	2	0		0	0,0	
		Zerendy region	village Sadovoye	58	39	17	0	2	0		0	0,0	
North Kazaksthan	2018	Ayirtau region	village Priozernoye	61	1	58	0	2	0		0	0,0	
		Musrepov region	village Novoishymskoye	17	1	16	0	0	0		0	0,0	
	2019	Musrepov region	region G.Musrepov	41	1	40	0	0	0		0	0,0	
			village Anreyevka	21	4	17	0	0	0		0	0,0	
			Village Nezhenka	23	2	19	0	2	0		0	0,0	
		Kyzylzharskiy region	region Kyzylzharskiy	21	0	21	0	0	0		0	0,0	
		Petropavlovsk city	city Petropavlovsk	40	0	40	0	0	0		0	0,0	
		Zhumabayev region	region Zhumabayev	34	1	33	0	0	0		0	0,0	
			village Konyuhovo	21	1	19	1	0	0		0	0,0	
Almaty Region	2019	Almaty city	Bauma's grove	7	7	0	0	0	0		0	0,0	
			Botanical garden	7	7	0	0	0	0		0	0,0	
			Bridge on the Ryskulov and Suyunbay str.	7	7	0	0	0	0		0	0,0	
			Bus station "70th travel"	38	38	0	0	0	0		0	0,0	
			Bus station "Lake"	2	2	0	0	0	0		0	0,0	
			Bus station "Stroykombinat"	44	44	0	0	0	0		0	0,0	
			Bus station "trading base"	28	28	0	0	0	0		0	0,0	
		Almaty region	Alma-Arasan	9	8	0	0	1	0		0	0,0	
			Along the Kapshagay highway	42	41	0	0	1	0		0	0,0	
			Butakovka	53	51	0	0	2	0		0	0,0	
			Kapshagay beach	43	43	0	0	0	0		0	0,0	
			Medeo	11	10	0	0	1	0		0	0,0	
			**Total**	**1058**	**575**	**456**	**6**	**21**	**8(0.8)**		**6**	**0,6**	

Supplement Table S2.: Overview of small mammals collection, sampling sites, captured numbers and numbers of positive specimen.

Region	Year	Sampling region	Sampling site	Rodents total	*Mus musculus*	*Clethrionomys glareolus*	*Meriones meridianus*	*Mircrotus arvalis*	*Microtus kirgisorum*	*Apodemus uralensis*	*Dryomys nitedula*	*Sorex spp.*	*Rattus norvegicus*	*Crocidura suaveolens*	OHFV positive of sampling site n (%)	OHFV in species
Almaty Oblast	2018	Almaty region	Bakanas	15	13	0	2	0	0	0	0	0	0	0	0	
			Tekeli	75	8	0	0	14	0	41	11	1	0	0	0	
		Almaty city	Flooplain small Almatinka	20	8	0	0	0	1	5	0	0	1	5	0	
			Stroikombinat	12	3	0	0	1	3	0	0	0	1	4	0	
			Railway	9	0	0	0	0	1	5	0	0	0	3	0	
			Airport	27	2	0	0	0	0	5	0	0	12	8	0	
			Mercur	12	0	0	0	0	0	9	0	0	0	3	0	
			Autopark	7	0	0	0	0	0	6	0	0	0	1	0	
	2019	Almaty region	Tekeli	79	6	0	0	28	0	43	2	0	0	0	0	
			Rudnichniy	30	0	0	0	30	0	0	0	0	0	0	0	
		Almaty city	Airport	35	6	0	0	0	2	5	0	0	19	3	0	
			Railway	39	9	0	0	0	22	6	0	1	0	1	0	
			Mercur	14	6	0	0	0	0	2	0	0	6	0	0	
			Tropinka	29	5	0	0	0	20	4	0	0	0	0	0	
West-Kazakhstan Oblast	2018	WKO	Bayterek	58	0	3	0	7	0	45	2	1	0	0	**1 (1.7)**	*A. uralensis*
			Borili	10	10	0	0	0	0	0	0	0	0	0	0	
			Oral	27	0	0	0	1	0	26	0	0	0	0	**1 (3.7)**	*A. uralensis*
			Taskala District	16	16	0	0	0	0	0	0	0	0	0	**2 (12.5)**	*M. musculus*
			Teretki	9	0	9	0	0	0	0	0	0	0	0	**3 (33.3)**	*C. glareolus*
	2019	WKO	Bayterek	46	10	0	0	0	0	36	0	0	0	0	0	
			Borili district	15	10	0	0	0	0	5	0	0	0	0	0	
			Oral	10	0	0	0	0	0	10	0	0	0	0	0	
			Teretki	27	16	0	0	5	0	6	0	0	0	0	0	
			**Total**	**621**	**128**	**12**	**2**	**86**	**49**	**259**	**15**	**3**	**39**	**28**	**7 (1.1)**	

Supplement Table S3.: Testing of Primers OHF-d1F and OHF-d2R and TaqMan probe targeting the E-Gen of OHFV against isolates of several Flavivirus and bacterial agents. Abbreviations: Reverse Transcriptase (RT), Tick-borne encephalitis (TBE), *Tick-borne encephalitis virus* (TBEV), information not available (n.a.).

**Agent**	**Strain**	**Familiy**	**Genus**		**Results of Realtime-RT PCR**
**Agents of family *Flaviviridae* , genus Flavivirus and part of the Tick-borne encephalitis (TBE)-serocomplex**					
Omsk hemorrhagic fever virus	*Bogoluva*	*Flaviviridae*	Flavivirus	TBE - complex	positve
Langat virus	*n.a.*	*Flaviviridae*	Flavivirus	TBE - complex	negative
TBEV Far Eastern subtype Sofjin	*Sofijn*	*Flaviviridae*	Flavivirus	TBE - complex	negative
Louping ill virus	*n.a.*	*Flaviviridae*	Flavivirus	TBE - complex	negative
**Agents of Family *Flaviviridae* , Genus Flavivirus**					
Dengue-1-virus	FGA/89	*Flaviviridae*	Flavivirus		negative
Dengue-2-virus	New Guinea-C	*Flaviviridae*	Flavivirus		negative
Dengue-3-virus	*CH53489*	*Flaviviridae*	Flavivirus		negative
Dengue-4-virus	*H241*	*Flaviviridae*	Flavivirus		negative
Zika virus	*H/PF/2013*	*Flaviviridae*	Flavivirus		negative
Yellow fever virus	*17DD*	*Flaviviridae*	Flavivirus		negative
West Nile virus	*NY99*	*Flaviviridae*	Flavivirus		negative
Bagaza virus	*n.a.*	*Flaviviridae*	Flavivirus		negative
Dakar bat virus	*n.a.*	*Flaviviridae*	Flavivirus		negative
Kedougou virus	*n.a.*	*Flaviviridae*	Flavivirus		negative
Spondweni virus	*n.a.*	*Flaviviridae*	Flavivirus		negative
Usutu virus	*n.a.*	*Flaviviridae*	Flavivirus		negative
**Other agents**					
Western equine encephalitis virus	*n.a.*	*Togaviridae*	Alphavirus		negative
Eastern equine encephalitis virus	*Ten Broeck*	*Togaviridae*	Alphavirus		negative
Herpes simplex virus 1,2	*n.a.*	*Herpesviridae*	Simplexvirus		negative
Varicella-zoster virus	*Ellen (ATCC-VR-1367)*	*Herpesviridae*	Varicellovirus		negative

* Primer sequences of OHF-d1F; OHF-d2R and TaqMan probe were taken from Ruzek *et al.* 2013.
